# Modulation of endothelium function by fatty acids

**DOI:** 10.1007/s11010-021-04260-9

**Published:** 2021-09-16

**Authors:** Rahul Mallick, Asim K. Duttaroy

**Affiliations:** 1 A.I. Virtanen Institute for Molecular Sciences, University of Eastern Finland, Kuopio, Finland; 2 Department of Nutrition, Institute of Basic Medical Sciences, Faculty of Medicine, University of Oslo, Oslo, Norway

**Keywords:** Endothelial cells, Atherosclerosis, CVD, FFA, LCPUFAs, Metaflammation, Prostaglandins

## Abstract

The endothelium acts as the barrier that prevents circulating lipids such as lipoproteins and fatty acids into the arterial wall; it also regulates normal functioning in the circulatory system by balancing vasodilation and vasoconstriction, modulating the several responses and signals. Plasma lipids can interact with endothelium via different mechanisms and produce different phenotypes. Increased plasma-free fatty acids (FFAs) levels are associated with the pathogenesis of atherosclerosis and cardiovascular diseases (CVD). Because of the multi-dimensional roles of plasma FFAs in mediating endothelial dysfunction, increased FFA level is now considered an essential link in the onset of endothelial dysfunction in CVD. FFA-mediated endothelial dysfunction involves several mechanisms, including dysregulated production of nitric oxide and cytokines, metaflammation, oxidative stress, inflammation, activation of the renin-angiotensin system, and apoptosis. Therefore, modulation of FFA-mediated pathways involved in endothelial dysfunction may prevent the complications associated with CVD risk. This review presents details as to how endothelium is affected by FFAs involving several metabolic pathways.

## Introduction

The tightly regulated vascular endothelium forms a vast interface between the flowing blood and neighboring tissues. The endothelium, which consists of a monolayer of endothelial cells, has a thickness of ≤ 1 μm and covers a total surface area of 4000 -7000 m^2^. Endothelial cells play various roles in the maintenance of vascularity of endothelium. Endothelial cells are responsible for a wide range of critical functions, including maintaining vascular tone, fluidity of blood, blood flow, and permeability of endothelium [1, 2]. In addition, the endothelium regulates normal functioning in the circulatory system by balancing vasodilation and vasoconstriction between thrombosis and hemostasis by modulating the several responses and signals [1, 3]. Thus, impairment of the endothelial functions plays deleterious roles in developing various disorders/diseases, including inflammatory angiitis syndrome, thrombotic embolism, disseminated intravascular coagulation disorder, neovascularization, tumor progression, and diabetic retinopathy [4].

The presence of atherosclerosis is a prevalent characteristic in cardiovascular disease (CVD), and that endothelial dysfunction is thought to be one of the early steps in the pathogenesis of atherosclerosis. Endothelial cells can affect various pathophysiological properties by producing different molecules [5]. Endothelium-secreted compounds such as angiotensin II (Ang II), endothelin-1 (ET-1), thromboxane(Tx) A_2_, and prostaglandin (PG) H_2_ involve in vasoconstriction, whereas nitric oxide (NO), prostacyclin (PGI_2_), bradykinin, and hyperpolarizing factor involve in vasodilation thus maintain a balance between the vasoconstriction and vasodilation [3]. Although dysregulated NO production is the main regulator of endothelium dysfunction, PGI_2_ and bradykinin also participate in regulating endothelium function. Therefore, the delicate balance between these endothelium-secreted molecules is critically required for the appropriate functioning of the endothelium [5]. Located at the interface between the circulation and the vessel wall, the endothelium reacts by synthesizing and releasing vasoactive substances with anti-thrombotic, vasodilating, and anti-atherogenic properties to maintain vascular homeostasis [6, 7].

The endothelium has a major function in regulating vascular tone, controlling blood flow, and blood fluidity and inflammatory responses [7, 8]. The inner layer of endothelium that comprises endothelial cells allows free blood flow and its cellular components. In addition, endothelial cells produce various factors regulating cellular adhesion, platelet aggregation response, smooth muscle cell proliferation, and inflammation [8, 9]. In addition, the endothelium exerts multiple actions on the expression of several pro-inflammatory genes, including those encoding adhesion molecules, chemokines, and other soluble cytokines of the endothelium [10–12].

Endothelial biology is affected by circulating lipids such as triglycerides-rich particles, chylomicron resident time, lipoproteins, and free fatty acids (FFAs) [13]. FFAs are released into the blood through the action of hormone-sensitive lipase on triacylglycerol stores in adipose tissue. Chylomicrons also contribute to plasma FFA levels, especially when high-fat diets are consumed. Increased plasma FFAs cause insulin resistance and endothelial dysfunction. The most observed lipid abnormalities in type 2 diabetes, metabolic syndrome, and CVD are hypertriglyceridemia, hyperlipoproteinemia, higher levels of FFAs, greater levels of small dense low-density lipoprotein (LDL) [14]. Several human epidemiologic and clinical intervention data suggested the association between circulating triacylglycerol levels and atherosclerosis [15, 16]. Plasma levels of FFAs, a well-established risk factor of CVD [17] are strongly associated with metabolic syndromes, obesity, and type 2 diabetes mellitus [18]. Several studies demonstrated that FFAs modulate the endothelial function and subsequent atherosclerosis processes. Elevated FFAs directly affect transcription factors of several genes involved in inflammation and oxidative stress in the endothelium.

FFA-induced endothelial dysfunction is mediated via several mechanisms, including impaired insulin signaling, dysregulation of NO synthesis, oxidative stress, inflammation, and the activation of the renin-angiotensin system (RAS) and apoptosis. Insulin resistance, oxidative stress, and inflammatory conditions are responsible for FFA-induced endothelium dysfunction [19–21]. Activation of endothelial cells by inflammation, proliferation, hypercoagulability, activation of vasoactive factors, enhanced apoptosis, increased vascular permeability, presence of free radicals is defined as endothelial dysfunction [22]. Because of the multifaceted roles of plasma FFAs in modulating endothelial function, elevated FFA level is now considered an essential link in the onset of endothelial dysfunction due to metabolic syndromes such as diabetes and obesity. Therefore, modulation of the signaling pathways involved in FFA-induced endothelial dysfunction may protect against endothelial dysfunction and subsequent CVD complications such as atherosclerosis.

Endothelial dysfunction is a significant feature in atherosclerosis, hypertension, diabetes, and cardiovascular disorders [7, 23]. Defective endothelium results in leukocyte adhesion, activation of platelets, pro-oxidation of mitogens, dysregulated synthesis of PGI_2_, NO, and endothelium-derived hyperpolarizing factor (EDHF), and other vasoconstriction factors such as Ang II and PGH_2_, resulting in atherosclerosis and thrombosis [24]. A balance between the levels of vasoconstrictors (TxA_2_, PGH_2_, ET-1) and vasodilators (NO, EDHF, PGI_2_) produced by the endothelium determines the vascular tone. The expression of adhesion molecules on endothelial cells also plays an important role in the atherosclerosis process. The adhesive molecules expressed on endothelial cells are selectins, intracellular adhesive molecules (ICAMs), and vascular adhesive molecules (VCAM-1).

This review aimed to describe the roles and fundamental mechanisms of FFA-mediated endothelial dysfunction. Moreover, how the endothelium participates in the uptake and metabolism of fatty acids and involves various biochemical pathways in health and disease is also discussed.

## Effects of fatty acids on endothelial function

Fatty acids are taken up and metabolized by endothelial cells, depending on the chain length, number, and position of double bonds. Fatty acids are beta-oxidized into mitochondria to fuel the tricarboxylic acid cycle and produce ATPs. At the same time, excess intracellular fatty acids are stored as cytosolic lipid droplets [25]. Protection from endoplasmic reticulum stress from excess FFA is correlated with endothelial stored lipid droplets [25]. Fatty acids are also synthesized de novo by endothelial cells even though they can uptake FFAs [26, 27]. Fatty acids are transported to mitochondria by the rate-limiting enzyme carnitine palmitoyltransferase 1a, the key to fatty acid oxidation and endothelial permeability [28, 29].

**Fig. 1:**
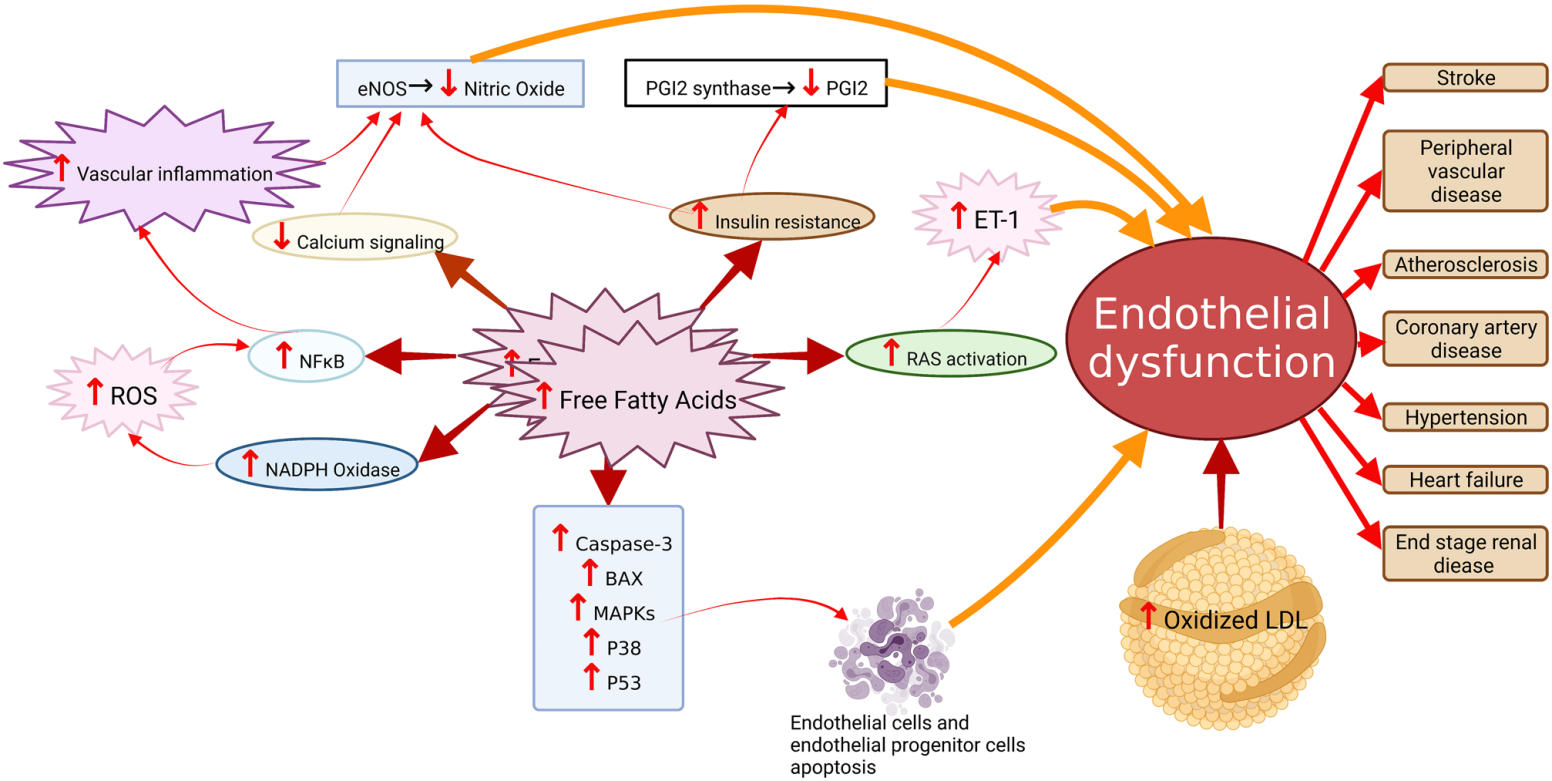
Mechanism of free fatty acids-induced endothelial dysfunction. Increased levels of free fatty acids reduce nitric oxide production directly or indirectly by mediating oxidative stress (ROS upregulation), upregulation of inflammatory signaling (NF-κB upregulation), and downregulation of insulin and calcium signaling. In addition, RAS activation by free fatty acids or oxidation of LDL can lead to vasoconstriction by ET-1 upregulation. Even apoptotic pathway activation by free fatty acids induces endothelial cells and endothelial progenitor cells apoptosis. Endothelial dysfunction ultimately leads to atherosclerosis events, coronary artery disease, hypertension, heart failure, peripheral vascular disease, stroke, and end-stage renal disease

High levels of blood FFAs affect endothelium function in different ways. First, FFAs may contribute to inflammation resulting in increased permeability of the endothelium. Second, elevated blood levels of FFAs also downregulate insulin-mediated production of NO and reduce blood flow peripherally. FFAs mediate these effects via two different mechanisms by (a) decreasing tyrosine phosphorylation IRS-1/2 and preventing the PI3K/Akt pathway, which regulates insulin-stimulated glucose uptake and NO production by endothelial nitric oxide synthase (eNOS) [18]. Figure 1 describes the mechanism of FFA-induced endothelial dysfunction.

In contrast, the endothelium-independent vasodilation remains unaffected [30], indicating that FFAs have specific inhibitory effects on NO synthesis by the endothelial cells. Infusion of FFA in insulin-sensitive human subjects was shown to significantly reduce NOS flux and impair shear stress-induced NO synthesis [31]. Palmitoylation of eNOS regulates the bioavailability of endothelial eNOS and thus NO release [32]. During atherosclerotic plaque formation, endothelial barrier, aberrant NO production, ROS production, and pro-inflammatory and pro-coagulative factors cause endothelial dysfunction, which enhances LDL transcytosis, upregulation of LDL oxidation, activation of blood platelet and leukocytes [33]. N-6 polyunsaturated fatty acids (PUFAs) may lower LDL cholesterol but increase LDL cholesterol susceptibility to oxidation and lower HDL cholesterol. Monounsaturated fatty acids are more beneficial than SFA and n-6 PUFAs in terms of their effects on LDL oxidizability [34]. They also prevented n-3 fatty acids from exerting their potential adverse effects of LDL oxidation. 15-Lipoxygenase is implicated in the in vivo oxidation of LDL and is a process thought to involved in initiation and progression of endothelium dysfunction and atherosclerosis.

More recently, inhibition of eNOS mRNA expression by FFAs was shown in rat aortic endothelial cells, thus lowering the eNOS activity and increased oxidative stress and inflammatory status [35]. FFAs downregulate insulin-mediated eNOS activity through upregulation of PTEN (phosphatase and tensin homolog) and simultaneous inhibition of Akt kinase [36]. The AMPK/PI3K/Akt/eNOS pathway in endothelium dysfunction was diminished with elevated levels of FFAs due to consuming a high-fat diet [37].

FFAs increase ROS levels in monocytes dose-dependently, leading to adhesion of the monocytes to the endothelial cells [38]—a critical mechanism in the development of atherosclerosis [39]. Furthermore, FFAs-induced increases in CVD risk factors, characterized by elevated endothelial markers, are also observed in healthy subjects [40]. Therefore, clinical and experimental studies exploit the protective effects of several dietary fatty acids/functional foods and drugs via suppression of inflammatory conditions and oxidative stress in FFA-induced endothelium dysfunction. As observed in human studies, calcium channel blockers such as dihydropyridine and amlodipine prevent FFAs-induced endothelium dysfunction, leucocyte activation, and oxidative stress. The protective effects of these drugs are mediated by the suppression of NF-κB p65 phosphorylation [41]. Moreover, these compounds involve in the nuclear localization of Nrf2 and reduce oxidative stress and enhance the expression of the cytoprotective genes in endothelial cells.

The RAS is one of the critical determinants of arterial blood pressure, and Ang II is a potent vasoconstrictor. The endothelial cell membrane expresses the angiotensin-converting enzyme (ACE) that produces Ang II. Ang II induces vasoconstriction by stimulation of endothelium and exhaustion of NO. Therefore, inhibition of ACE stimulates vasodilation via the NO pathway [42]. While Ang II increases FFAs levels via downregulating the fatty acid oxidation [43], on the other hand, FFAs also activate the RAS system [44]. ACE^−/−^ mice had increased expression of genes responsible for lipolysis and oxidation of fatty acids [45], explaining the possible interplay between RAS and FFAs. By activating leukocytes, thus FFAs contribute to the adhesion of leukocytes to the endothelium in an Ang II-dependent manner, leading to the initiation of endothelium dysfunction [46]. Consequently, inhibition of the RAS prevents the FFA-induced endothelium dysfunction [47]. A single dose of either losartan (an Ang II receptor antagonist) or perindopril (an ACE inhibitor) completely prevented the FFAs-induced dysregulation of endothelium-dependent vasodilation, suggesting the blockade of RAS as an effective treatment for FFAs-induced endothelium dysfunction [47].

The risk of CVD is associated with saturated fatty acids (SFAs). In contrast, unsaturated fatty acids are unlikely involved in CVDs, and instead, these fatty acids are mostly found beneficial in CVD. SFAs increase blood LDL levels, a significant risk factor for CVDs [48]. Studies have demonstrated that long-chain saturated fatty acids(c > 14) (LCFAs) impart a greater risk for CVD than by the medium-chain fatty acids(c8-c12) (MCFAs) [48]. The most common saturated LCFAs present in the western diet are myristic acid,14:0 (MA), palmitic acid, 16:0 (PA), and stearic acid,18:0 (SA). While SFAs have been shown to increase total cholesterol and LDL cholesterol, there is also an increase in high-density lipoprotein (HDL) cholesterol [49]. CVD risk associated with SFAs varies from no association to a significantly significant risk [50]. SFAs, depending on chain length, have diverse effects on cholesterol metabolism in the body. For example, stearic acid, 18:0 (SA), is considered neutral on cholesterol metabolism compared with the other long-chain SFAs due to its high conversion rate to oleic acid 18:1n-9 (OA) by Δ9-desaturase enzyme. Long-chain SFAs, the health impact of stearic acid has no harmful effect on CVD risk. SFAs exert their atherogenic and thrombogenic influence through increased production of VLDL particles and ApoA1, decreasing LDL receptors-specific activity and increased platelet aggregation [51]. Unlikely other SFAs, SA showed no atherogenic or thrombogenic effect since, after absorption, it is desaturated to monounsaturated fatty acid, OA, which is associated with a beneficial effect on cardiovascular health. OA is incorporated into phospholipids rather than into triglycerides and cholesterol esters. OA exerts significant beneficial effects on atherosclerosis and thrombosis [52–54].

Various studies have concluded that PA substantially contributes to the development of atherosclerosis [55]. Both in vivo and in vitro studies have demonstrated the mechanisms by which PA contributes to the pathogenesis of CVDs. PA promotes inflammatory responses and cellular senescence in cardiac fibroblasts. PA achieves senescence in these cells by activating toll-like receptor 4 (TLR4) and NLRP3 inflammasome, increasing mitochondrial ROS levels [56]. PA also induces apoptosis of the vascular smooth muscle cells by inducing the TLR4 pathway and generating ROS [57]. Both PA and SA downregulated eNOS in porcine aortic endothelial cells. Although PUFAs have a protective role against endothelium dysfunction [58], their elevated FFAs adversely affect the endothelium by decreasing NO release and increasing ET-1 levels. Linoleic acid,18:2n-6 (LA) negatively regulates eNOS phosphorylation and affects the intracellular NO levels in ECV304 cells [59].

Many clinical studies showed protective effects of OA on flow-mediated dilation and other endothelial markers; however, they did not focus on FFA-induced endothelium dysfunction [60–62]. Studies demonstrated that n-3 long-chain polyunsaturated fatty acids (LCPUFAs) such as docosahexaenoic acid, 22:6n-3 (DHA), eicosapentaenoic acid,20:5n-3 (EPA), and polyphenols were beneficial in FFA-induced endothelium dysfunction [59, 63–65]. The mitochondria-related AMPK/eNOS pathway alleviates endothelium dysfunction and atherosclerosis in mice fed with a high-fat diet [66]. L-carnitine, an essential factor for fatty acid transport/oxidation in the mitochondria, attenuates FFA-induced obesity-related endothelium dysfunction in human subjects [67].

Moreover, SA showed a similar reducing impact on platelet aggregation and coagulation factors as the OA and LA [68]. Besides, a high-fat diet, a significant source of SFAs in blood levels of FFAs, induces oxidative stress in endothelium [69]. Elevated FFAs induce expression of NADPH and mediates oxidative stress in rats with characteristics of both obesity and type 2 diabetes mellitus [70]. FFAs-induced endoplasmic reticulum stress was observed in endothelial cells isolated from healthy human subjects [71]. Infusion of intralipid in healthy subjects increased FFAs by a 4.2-fold increase, and that was associated with reducing the hyperemic response in the leg without a change in flow-mediated dilation of the brachial artery. The mRNA levels of the genes ATF6 and IRE1, responsible for early adaptive responses to endothelium reticulum stress, increased in endothelial cells.

*Trans* fatty acids (TFAs), obtained from partial hydrogenation of plant oils, have the highest atherogenic activity. Thus industrial foods—cakes, cookies, and crackers often contain a high content of TFAs. TFAs have one double bond in which the hydrogens are on the opposite side to one another resulting in physiochemical properties close to those of SFAs. Main TFAs are elaidic acid, 18:1n-9t, and trans-vaccenic acid, 18:1n-7t. TFAs are associated with a higher risk of CVD and type 2 diabetes mellitus [72]. Consumption of TFAs increased plasma LDL cholesterol, lowered HDL cholesterol, and increased lipoprotein (a) and plasma triglyceride levels [73] and smaller flow-mediated vasodilation [74]. TFAs can influence the thrombotic state through the eicosanoid synthesis pathway [72]. TFAs also adversely affect endothelial function, which partly explains their association with CVD risk [75]. Intake TFAs also induced inflammation and endothelial dysfunction, as evidenced by increased plasma levels of CRP, IL-6, soluble tumor necrosis factor receptor (TNF-2), E-selectin, and soluble intercellular and vascular cell adhesion molecules (sICAM-1 and sVCAM-1) in apparently healthy women [75]. TFAs also activate the NF-κB pathway that increases endothelial superoxide production and reduces NO synthesis [76]. FFAs-induced endothelium dysfunction is mediated via the activation of NF-κB [77]. Thus, the NF-κB pathway is a significant player mediating the harmful effects of TFAs on human coronary artery endothelial cells [78].

FFAs-induced NLRP3 inflammasome also increases endothelial permeability. PA activates the NLRP3 inflammasome with a resulting decrease in endothelial tight junction proteins—zonula occludens-1 and -2 in microvascular endothelial cells. FFAs also increases the synthesis of high-mobility group box 1 (HMGB1) protein, which might explain the early onset of endothelial injury during obesity by FFAs.

Endothelial progenitor cells (EPCs) participate in endothelial recovery following arterial injury and oxidative stress-induced damages [79]. Dysfunctional EPCs are involved in the pathogenesis of CVD [80]. PA contributes to the apoptosis of EPCs mediated via the p38 and JNK/MAPKs pathways [81]. PA has harmful impacts on EPCs in metabolic syndrome patients via regulating long non-coding RNA (LncRNA) maternally expressed gene 3 (MEG3) [81]. These data raise interest for further studies on the pathophysiological roles of MEG3 in both EPCs and endothelial cells.

## Metabolism of n-3 and n-6 long-chain polyunsaturated fatty acids and their impacts on endothelium

Dietary polyunsaturated fatty acids (PUFAs) commonly consumed by humans encompass two major groups: the n-3 and n-6 families of fatty acids. Linoleic acid, 18:2n-6 (LA) and alpha-linolenic acid,18:3n-3 (ALA) are the dietary essential fatty acids (EFAs) [82]. LA and ALA are not interchangeable but can be further elongated and desaturated by the same enzyme systems to produce n-6 and n-3 LCPUFAs in the body. Some common n-3 PUFAs include ALA, EPA and DHA, and common n-6 ones include LA and arachidonic acid,20:4n-6 (ARA). LCPUFAs are the precursors for eicosanoid biosynthesis and various signaling compounds with relevant roles in human health and disease. Whereas dietary LA and ALA are primarily from vegetable oils, preformed LCPUFAs may also be consumed in animal-origin foods. The importance of LCPUFAs has been related to their structural action, their specific interaction with membrane proteins, and their ability to serve as precursors of second messengers. LCPUFAs (20 carbon) are substrates for cyclooxygenase (prostaglandin-endoperoxide synthase) and lipoxygenases and produce various compounds collectively called eicosanoids. These compounds have diverse biological functions in cell growth and development, inflammation, and the cardiovascular system. The biological response elicited after eicosanoid release is dependent on the net balance of eicosanoids derived from n-6 and n-3 LCPUFAs. ARA is the most predominant precursor fatty acid of the highly biologically active eicosanoids of the 2 series in the Western diet. Also, the endogenous formation of cyclooxygenase and non-cyclooxygenase metabolites of fatty acids has been implicated in gene expression. Because each type of EFA can interfere with the other's metabolism, an excess of n-6 fatty acids will reduce the metabolism of ALA, possibly leading to a deficit of n-3 LCPUFA metabolites. Therefore, a proper balance between the n-6 and n-3 fatty acids in the diet is essential to maintain optimum health.

Endothelial cells metabolize ARA in three different ways, cyclooxygenase (COX), lipoxygenase and, cytochrome P450 system. The primary metabolites of the COX pathway are PGI_2_ with lesser amounts of PGE_2_, TxA_2_, and 12-hydroxyheptadecatrienoic acid [83]. 12- and 15-hydroxyeicosatetraenoic acids are the primary lipoxygenase metabolites. Endothelial cells synthesize the four regioisomeric epoxyeicosatrienoic acids (EETs), 14,15-, 11,12-, 8,9-, and 5,6-EETs, with 14,15- and 11,12-EETs the significant metabolites.EETs are cytochrome P450 epoxygenase metabolites of ARA. PGE_2_ and TxA_2_ play an essential role in maintaining vascular homeostasis [83]. PGI_2_ is a vasodilator and a platelet aggregation inhibitor, whereas TxA_2_ is a vasoconstrictor and activator/aggregator of blood platelets [84, 85]. PGI_2_ has vasodilating properties, inhibits platelet aggregation by elevating intracellular levels of cAMP [86]. At the same time, vasorelaxation and platelet inhibitory actions of NO are mediated predominately by activating intracellular guanylyl cyclase, leading to cGMP formation [8]. Thus, PGI_2_ and TxA_2_ play a critical role in maintaining vascular homeostasis. PGI_2_ is a vasodilator and an inhibitor of platelet aggregation, whereas TxA_2_ is a vasoconstrictor and a promoter of platelet aggregation [86, 87]. Therefore, an imbalance in PGI_2_ or TxA_2_ production is implicated in the pathophysiology of many thrombotic and cardiovascular disorders [83, 87]. The protective effect of PGI_2_ against the development of CVD is mediated through the inhibition of various cellular processes, including inhibition of platelet activation, leukocyte adhesion to endothelium. Prostaglandins also enhance endothelial cell survival through the upregulation of the anti-apoptotic protein B cell lymphoma (Bcl)-2 [88] and the activation of phosphatidylinositol (PI)3-kinase (K)-Akt pathway [89].

N-6 LCPUFAs mediate acute inflammatory responses by synthesizing pro-inflammatory eicosanoids [90], whereas n-3 LCPUFAs produce anti-inflammatory or neutral eicosanoids. ARA-derived eicosanoids control cellular membrane lipid composition, inflammation, coagulation, and vascular homeostasis [91, 92]. Also, ARA-induced synthesized cytokines and adipokines play a vital role in metabolism and inflammation [93]. LA induces inflammation by increasing the levels of TNF-α, MCP-1, VCAM-1, and ICAM-1 through the activation of NF-κB and activator protein 1 (AP-1) [94], and it affects the release of NO [95].

Circulating blood platelets do not attach to the negatively charged surface of the endothelium. However, the activated platelets can bind GpIbα to either P-selectin or VWF on the surface of the endothelium, and indirectly via a fibrin bridge that joins GpIIb/IIIa and ICAM-1. Whereas NO decreases the intracellular level of Ca^2+^, the transformation of GPIIb/IIIa platelet receptor and suppresses the integrin's binding to fibrinogen [96, 97]. The ecto-ADPase (CD-39), on the surface of endothelial cells, hydrolyzes both ATP and ADP to generate AMP, thus decreases platelet aggregation/activation [86, 96]. TxA_2,_ produced by the endothelium from ARA, aggregates platelets and expresses adhesive co-factors for platelets such as vWF, fibronectin, and thrombospondin procoagulant factors such as factor V [84, 98].

Endothelial cells actively prevent thrombus formation by suppressing platelet adhesion and activation [99]. The vasoprotective function of endothelial cells is also associated, among others, with biosynthesis and release of NO, PGI_2_, PGE_2_, and tissue plasminogen activator (tPA). Platelet activation is counteracted by PGI_2_ and PGE_2_, produced from ARA by the endothelium after activation by various vasoactive agents, including thrombin. PGI_2_ is produced by the endothelium of large vessels, while the endothelial cells from smaller vessels produce PGE_2_ [100]. The effect of PGI_2_ is enhanced by NO produced by endothelial nitric oxide synthase (eNOS). The endogenous fibrinolytic system is responsible for the dissolution of the thrombus. It is regulated by the endothelium-derived profibrinolytic factor, tissue plasminogen activator (tPA), and its inhibitor, plasminogen activator inhibitor type-1 (PAI-1) [101]. Fatty acids, depending on their structure, regulate PAI-1 and tPA activity. However, the definitive conclusions are yet to be reached [102–104]. These endothelium-derived compounds can inhibit activation of platelets and leukocytes, promote fibrinolysis, maintain tissue perfusion and protect the vascular wall against acute damage and chronic remodeling. Endothelial dysfunction is associated not only with suppression in the release of these compounds but also with the secretion of deleterious compounds such as PGH_2_, PGG_2_, superoxide anion (O_2_-, peroxynitrite (ONOO-), and plasminogen activator inhibitor (PAI-1).

Several epidemiological, experimental, and clinical studies indicate that n-3 fatty acids can decrease CVD risk via several mechanisms, including improving vascular function. Incorporation in cell membranes, n*-3* PUFAs precisely activate cardiovascular protective signaling pathways [105], increase NO production [106], reduce oxidative stress [107] and inflammation [12]. N*-3* fatty acids also increase eNOS expression in the endothelium via several pathways such as phosphorylation of AMPK [63] and increased expression of eNOS mRNA [108]; stimulation of SIRT-1 and heat-shock protein 90 protein [109, 110]; and finally, eNOS translocation from caveolae to the cytosol [111]. N-3 PUFAs can decrease the expression of these adhesive factors of endothelial cells and thus reduce the activation of the endothelium.

The vasoprotective properties of n-3 fatty acids include decreased arterial plaque formation, anti-inflammatory activity, improved endothelial-dependent vasodilation, lowered arterial blood pressure, and increased antioxidant activity. N-3 fatty acids reduce arterial stiffness and blood pressure via eNOS expression.[112, 113]. Monocyte-endothelium interaction is essential in many acute and chronic inflammatory diseases. Both EPA and DHA significantly reduce PAF synthesis, monocyte rolling, and adherence, whereas endothelial adhesion molecules expression was unaltered.

Both EPA and DHA compete with ARA as substrates to form pro-inflammatory mediators, such as 4 series of leukotrienes, 2 series of prostaglandins, and cytokines. In addition to competitive inhibition of the *n* -6 fatty acid pathway, *n*-3 fatty acids also inhibit the production of C reactive protein, TNFα, matrix metalloproteinase (MPP)-2 and MPP-9, and tissue inhibitors of MPP [114–116]. Furthermore, N-3 fatty acids inhibit COX-2 expression as therapeutic potential as COX-2 overexpression is involved in different inflammatory/degenerative diseases apart from atherosclerosis [112].

Endothelial cells express ICAM-1, VCAM-1, E-selectin, and P-selectin involved in leukocyte recruitment and platelet adhesion during thrombosis and inflammation and contribute to early phases of atherosclerosis. Cytokine-induced endothelial activation involves increased expression of genes for ICAM-1, VCAM-1, and E-selectin, whereas n-3 PUFAs suppress the synthesis of inflammatory cytokines that activate the endothelium. Thus, n-3 PUFAs decrease atherosclerosis and inflammation by reducing the expression of adhesion and migration of monocytes to the endothelium.

N-3 LCPUFAs supplementation replaces ARA content in plasma membrane phospholipids to improve endothelium vasodilating effects [117]. Thus, n-3 PUFAs can modify n-6 eicosanoids production to favor vasodilation and anti-thrombotic actions. N-3 PUFAs increase endothelium-dependent relaxation by increased release of NO. NO inhibits platelet aggregation and adhesion, leukocyte adhesion, and smooth muscle cell proliferation. The protective role of n-3 LCPUFAs has been shown by reducing FFA-induced endothelium dysfunction via the AMPK/PI3K/Akt/eNOS pathway. EPA has a protective role against PA-induced endothelium dysfunction mediated via activation of the AMPK/eNOS pathway. The EPA also inhibited PA-induced apoptosis of endothelial cells and activated apoptosis-related proteins, such as caspase-3, p53, and Bax [63]. N-3 PUFAs can protect endothelium by reducing platelet TxA2 synthesis, plasminogen activator inhibitor-1 activity, COX 1 or 2 productions, platelet activation, and adhesion to endothelium [118–121]. Moreover, n-3 PUFAs interfere with the vitamin K-dependent carboxylation of coagulation factors II, VII, IX, and X) [122, 123].

Dietary n-3 PUFAs reduce plasma triglycerides and very-low-density lipoproteins (VLDL) [124–126] and thus protect the endothelium from lipid stress. Inhibition of 1,2 diglyceride acyltransferase or sterol regulatory element-binding protein 1c by n-3 PUFA reduces hepatic triglyceride synthesis [127–129]. Also, n-3 PUFA activates peroxisome proliferator-activated receptor alpha (PPARα) to favor catabolism of circulating triglycerides and VLDL by promoting fatty acid beta-oxidation [127, 130]. N-3 PUFA induced non-proteasomal degradation of apolipoprotein B reduces naïve hepatic VLDL secretion [131–133]. Several studies found that n-3 PUFAs increase HDL levels despite fluctuating effects on LDL levels, possibly by decreasing cholesteryl ester transfer protein activity [134–138].

As an independent risk factor of CVDs, hypertension can be favorably regulated by n-3 PUFAs supplementation [139, 140]. N-3 PUFAs mediated blood pressure regulation by producing vasodilator prostaglandins, activating eNOS, incrementing renin release from the kidney, suppressing ACE activity, and activating large-conductance Ca^2+^-activated K^+^ channels has been demonstrated [141–144]. In addition, studies found that reduced n-3 PUFAs on red blood cells induce vasoconstriction and increased production of pro-inflammatory eicosanoids [145, 146].

## Fatty acid transport across the endothelium

Fatty acids pass through the endothelial cell membrane to enable tissue uptake in muscle and likely allow fatty acids to penetrate the arterial wall. The trans-endothelial crossing could take place through movement around or between endothelial cells. Large topical concentrations of FFAs disrupt the endothelial barrier, as does active lipolysis, which can also upregulate LDL passage into the artery [147]. Fatty acid uptake by endothelial cells is not entirely understood clearly; it might involve both protein-mediated uptake and non-specific uptake [148], which might occur in the presence of high topical fatty acid concentrations, e.g., those that occur during chylomicron but not VLDL lipolysis [149]. Fatty acid metabolism by vascular endothelial cells occurs both under basal conditions and following endothelial cell stimulation or injury.

Endothelial cell reigns in the metabolic homeostasis of the various tissues via their growth and function. Endothelial cell activates in response to vessel wall permeability and interaction with multiple molecules of blood. However, glucose is the primary source of endothelial energy, fatty acid uptake, transport, and metabolic pathways on endothelial cells have become an arena of interest for many years [150]. As the metabolic gatekeeper, endothelial cells regulate fatty acid transcytosis, and dysregulated endothelial fatty acid transport can pronounce insulin resistance, which plays a pivotal role in various pathological processes [151].

**Fig. 2:**
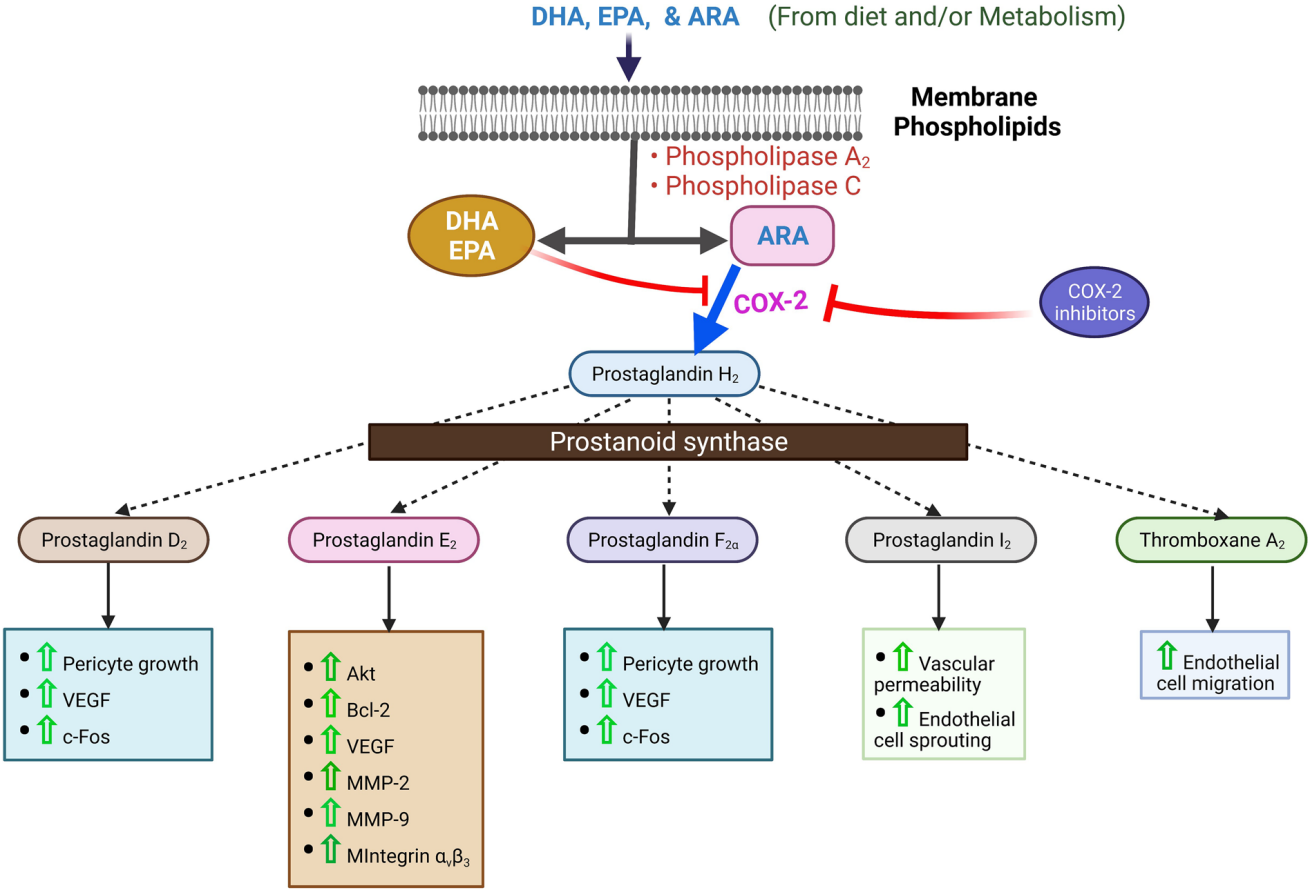
Rate limiting the prostanoid-induced inflammatory angiogenic role of COX-2 on endothelial cell. N-3 or N-6 LCPUFA6 (DHA, EPA and ARA) from dietary or metabolism are incorporated into cell membrane phospholipids. DHA, EPA, or ARA can be liberated by phospholipase A_2_ or phospholipase C. COX-2 then converts ARA to prostaglandin H_2_, which can be inhibited by the liberated DHA, EPA, or COX-_2_ inhibitors. Prostaglandin H_2_ converts into prostaglandin D_2_, prostaglandin E_2_, prostaglandin F_2_α, prostaglandin I_2,_ and thromboxane A_2_ by prostanoid synthase to promote-specific angiogenic steps and mediators

Endothelial fatty acid transport can be either passively by crossing paracellularly through intercellular space between neighboring endothelial cells or actively, where fatty acids cross the endothelial layer by different transporters such as plasma membrane fatty acid-binding protein (FABPpm), fatty acid transport proteins (FATPs), cluster of differentiation 36 (CD36), or fatty acid translocase (FAT), and cytoplasmic fatty acid-binding proteins (FABPs) [152]. Unlike fenestrated hepatic endothelial lining, continuous and non-fenestrated cardiac endothelial lining signifies the importance of the endothelial fatty acid transport system. Unlike medium and short-chained fatty acids, generally consumed long dietary chain (c ≥ 14 carbons) fatty acid uptake in endothelial cells is upregulated by synergistic co-expression of *FATP3* and *FATP4*. Secretion of vascular endothelial growth factor B by cardiomyocytes or production of 3-hydroxy-isobutyrate by skeletal muscle regulates the expression of *FATP3* and *FATP4* in neighboring endothelial cells [27, 153]. *FATP4* and *FATP5* are mainly expressed by capillaries and venules of the heart, skeletal muscle, and adipose tissue [154]. Apelin signaling within endothelial cells inhibits *FATP4* expression by activating transcription factor *FOXO1*, which may stabilize VE-cadherin-mediated endothelial cell junctions [155, 156]. In cardiac and skeletal muscle, deficiency of fatty acid uptake is compensated by glucose uptake for energy production [154]. Figure 2 describes the prostanoid-induced angiogenic role of COX-2 in endothelial cells.

FAT/CD36 facilitates fatty acid transport across the endothelial layer and oxidation in the heart, skeletal muscle, and adipose tissue [157, 158]. Silencing FAT/*CD36* or lipoprotein lipase downregulates peroxisome proliferator-activated receptor alpha mediated cardiomyocyte lipid deposition, ultimately resulting in lipotoxic cardiomyopathy [159]. Surprisingly the opposite effect of FAT/CD36 is observed in the liver, which may be due to the high permeability of hepatic endothelial cells comparatively [160]. As the principal regulator of fat metabolism, peroxisome proliferator-activated receptor-gamma ((PPARγ) maintains different fatty acid transport proteins expression, including FAT/CD36 in nonfenestrated endothelium [161]. Ultimately fatty acid transport, storage, and release in heart, skeletal muscle, and adipose tissues solely depend on PPARγ [162].

Surprisingly, when fatty acid level crosses the metabolic limit, FAT/CD36 becomes dysfunctional and consistently stays at the plasmalemma [163]. Although n-3 PUFAs downregulate the ongoing relocation of FAT/CD36 on sarcolemma and prevents insulin resistance [164]. Different factors control post-translational modification of FAT/CD36, such as glycosylation, ubiquitination, acetylation, palmitoylation, etc. [165]. A better understanding of these processes will help us to understand FAT/CD36 trafficking in the cells.

Overexpression of FAT/CD36 upregulates fatty acid oxidation around three-fold [166]. FAT/CD36 works as the ferry for disposing fatty acids into mitochondria. Mitochondrial co-localization of FAT/CD36 is proportionate to the amount of fatty acid oxidation [167]. FAT/CD36 can solely upregulate fatty acid esterification and/or lipoprotein packaging [168]. FAT/CD36 seems to be interrelated with AMPK. FAT/CD36 -induced AMPK increases fatty acid oxidation, while AMPK induces FAT/CD36 on the cell membrane [163, 169, 170]. FAT/CD36 inhibits AMPK by allowing tyrosine kinase Fyn to block AMPK-kinase LKB1 reversely without fatty acid. These coordinated functions can be dysregulated by interrupted AMPK signaling due to FAT/CD36 deletion [171–173].

Also, FAT/CD36 regulates cytosolic Ca^2+^ and Ca^2+^-dependent phospholipase A2 activation [174]. Either interruption of Sarco/endoplasmic reticulum calcium ATPase (SERCA) or following reduction of Ca^2+^, endoplasmic reticulum Ca^2+^ sensor Stromal interaction molecule 1 stimulates membrane store-operated Ca^2+^ channels (Orai protein multimers) to maintain cytosolic Ca^2+^ [175]. FAT/CD36 regulates store-operated Ca^2+^ channels, which are vital for maintaining myocardial health. FAT/CD36 knockdown interrupts cytosolic Ca^2+^ clearance, which upregulates SERCA2 as compensation. Conduction anomalies, e.g., bradycardia, atrioventricular blockage, are obvious following FAT/CD36 knock-down, while tachyarrhythmogenic effects are caused by FAT/CD36 overexpression [173, 176]. Although FAT/CD36 overexpressed heart reduces infarction size following infarction [176]. Myocardial remodeling is influenced by FAT/CD36 mediated Ca^2+^-dependent phospholipase A2 activation [174].

Angiogenic regulator Notch has also shown a predominant role in FABP4, FABP5, lipoprotein lipase, FAT/CD36, and angiopoietin-like 4 (ANGPTL4) expression [177]. Ultimately, endothelial Notch signaling interrupts insulin sensitivity and glucose tolerance [178]. Even current finding has shown the pivotal role of another angiogenic regulator, angiopoietin 2 (ANGPT2), in modulating FAT/CD36 and FATP3 expression through integrin α5β1 signaling and key to insulin resistance [179]. All these findings demand to investigate more precisely the relationship between angiogenesis and fatty acid transport.

It is noteworthy that fatty acid transport and intravascular lipoproteins transport occur on the apical endothelial surface. For example, approximately 30% of LDL is metabolized by fenestrated endothelium, but LDL receptors and CD36 are crucial for LDL internalization [180, 181]. On the other hand, high-density lipoprotein is known as good lipoprotein transcytoses by several transporters, such as ATP binding cassette transporter G1, A1, and scavenger receptor class B type I. But it’s still obscured if triglyceride can cross the endothelium or not. Again glycosylphosphatidylinositol-anchored high-density lipoprotein-binding protein 1(GPIHBP1)-dependent lipoprotein lipase is transported from adipocytes or myocytes to endothelium for hydrolysis of triglyceride and lipolysis of chylomicrons [182, 183].

Cytoplasmic FABPs are highly expressed in metabolically active tissues [184]. FABPs are responsible for metabolic homeostasis and different lipid-mediated biological processes in various tissues despite the similarities in their tertiary structures and ligand affinity [152, 185]. Metabolic disorders play a crucial role in CVDs, abnormal lipid signaling, high lipids storage, trafficking, and signaling capacity of macrophages and adipocytes. Henceforth, the contribution of FABPs in lipid-related physiology and cardiovascular impacts is significant [184, 186]. However, the biological activities of FABPs remain a mystery [152]. Different studies have found that endothelial-FABP4 expression is upregulated in various organs [187–189]. The pro-angiogenic role of endothelial-FABP4 is induced by promoting cell proliferation, survival, and migration [188–190]. FABP4 also regulates the functions of various mitogenic pathways, such as stem cell factor/c-kit signaling, which plays a vital role in its pro-angiogenic activity. In turn, FABP4 expression is controlled by vascular endothelial growth factor-A (VEGF-A) and mammalian endothelial target of rapamycin complex 1 [188, 189]. FABP4 also determines the inflammatory function of endothelial cells by regulating the gene expression that plays crucial roles in endothelial cell activation, including endothelial eNOS and intercellular adhesion molecule 1.

FABP5 has a 55% amino acid sequence homology with FABP4 and primarily microvascular expression pattern in endothelial cells [154, 191]. Although, the FABP5 function in endothelial cells remains unclear. The current research has found the vital role of FABP4 and FABP5 in fatty acid uptake in the heart and skeletal muscle endothelial cells [154]. In animal models, combined deficiency of FABP4 and FABP5 ensures more excellent defence against insulin resistance, obesity, and atherosclerosis than mice deficient for either FABP4 or FABP5 [192, 193]. Along with LCFAs, FABP5 binds with PPARd and retinoic acid; upon binding these ligands, it mobilizes to the nucleus to induce PPARd and regulates cell growth and development. Regulation of endothelial cells homeostasis and vascular integrity is vital to organ physiology and tissue repair, regeneration, and tumor growth. Based on the additive functions of FABP4 and FABP5 in other cell types and their co-expression in microvascular endothelial cells, FABP5 played a role in regulating angiogenesis-related endothelial cell functions, including proliferation, migration, and survival. As a downstream inducer of FABP5-related effects in endothelial cells, the critical role of PPARδ was demonstrated.

The expression of FABP3 and FABP5 has been demonstrated in the microvascular of cardiac tissues and skeletal muscles. Also, FABP4 and FABP5 expressions were figured out in the microvessels of other organs with higher fatty acids metabolic activity, such as hepatic and adipose tissues [194, 195]. Endothelial cells can regulate the expression of FABP5 depending on tissue types and microvasculature [191]. Further investigations are needed to figure out the exact role of FABPs in lipid metabolism.

## Roles of fatty acid metabolism in endothelial cells on angiogenesis

The metabolism of endothelial cells has only recently been recognized as a driving force of angiogenesis. Metabolic pathways, such as glycolysis, fatty acid oxidation, and glutamine metabolism, have distinct, essential roles during vessel formation. Moreover, endothelial cell metabolism is markedly perturbed in pathologies such as cancer and diabetes. For instance, because tumor endothelial cells increase glycolysis, lowering hyper glycolysis in tumor endothelial cells induces therapeutic benefits in preclinical tumor models. Expanding our knowledge of how endothelial cells alter their metabolism in disease could pave the way for novel therapeutic opportunities. The most recent data describe the association of endothelial cell metabolism in health and disease, emphasizing the changes in metabolism in the tumor endothelium.

The endothelial lining on the vessel wall is crucial for nutrients and oxygen supply to the tissue. Reduced nutrients availability or oxygen tension stimulates activation of quiescent endothelial cells for angiogenesis (de novo blood vessel formation from pre-existing blood vessels) [196, 197]. Angiogenic sprouting involves interaction between endothelial cells and their metabolic microenvironment. First, proangiogenic proteins are secreted from the oxygen and nutrition demanding tissues to activate endothelial cells. Activated endothelial cells extend filopodia (known as tip cells) towards angiogenic stimuli [198]. Migratory tip cells are followed by proliferating endothelial cells (known as stalk cells) to elongate the sprouting process [199]. Endothelial cells continuously compete to take the lead as tip cells [200]. Ultimately tip cells communicate with tip cells of adjunct sprouts to form de novo vascular circuits throughout the process until meeting oxygen and nutritional demands. Then proangiogenic factors downregulate, basement matrix establishes, pericytes recruit to onset perfusion, and endothelial cells become quiescent again. These endothelial cells of de novo blood vessels are defined as phalanx cells [201].

**Fig. 3:**
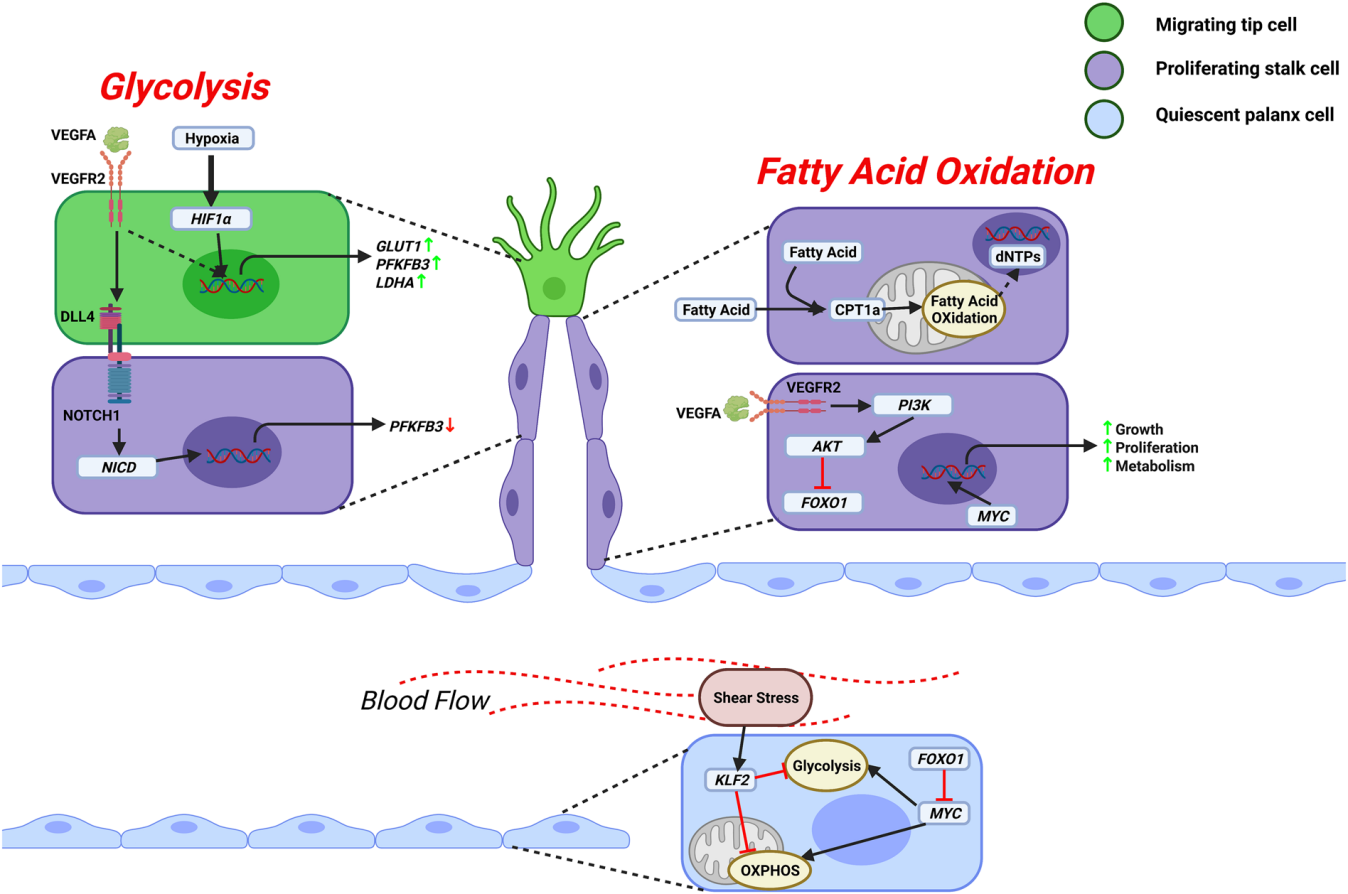
Regulation of endothelial metabolism. VEGFA induces *LDHA, PFKFB3, GLUT1,* etc., through the VEGFR2 mediated glycolysis pathway to support tip cell migration. Hypoxia can also promote glycolysis by inducing *HIF1α.* Glycolysis also supports stalk cell proliferation by downregulating DLL4-NOTCH1 signalling-dependent *PFKFB3* gene expression. Furthermore, even VEGFA blocks growth-inhibiting transcription factor *FOXO1* via VEGFR2 mediated PI3K/AKT pathway to support the proliferation of stalk cells. Also, growth-enhancing transcription factor *MYC* promotes growth, anabolic metabolism, and proliferation of stalk cells. Along with glucose, proliferating stalk cells consume fatty acids, contributing to nucleotide synthesis for cellular proliferation. But in quiescent phalanx cells, *FOXO1* represses *MYC* signaling to reduce glycolysis and mitochondrial metabolism. In addition, turbulent blood flow-induced shear stress-mediated activation of transcription factor *KLF2* leads to reduced metabolic rate

Angiogenesis requires coordinated not only endothelial morphogenic behavior but also metabolic activities. The metabolic activities of endothelial cells are different from other differentiating cells. Rapid energy production is needed for tip cell migration and navigation, while proliferative stalk cells need to produce cellular components [202]. Like cancer cells, around 85% energy production of endothelial cells depends on the aerobic glycolytic pathway [203–205]. Accelerated glycolysis by VEGFA increases endothelial glucose uptake and upregulation of glycolysis activators. Moreover, VEGF induces endothelial cell proliferation through the upregulation of FABP4 [190]. Figure 3 describes the regulation of endothelial metabolism by different factors. Also, VEGFR2 and NOTCH1 activities rely on glycolysis [206, 207]. But hypoxia-induced HIF1α can also activate glycolytic genes, e.g., *SLC2A1, LDHA, PFKFB3* [208–210]. PFKFB3 driven glycolysis is vital for tip cell migration and stalk cell proliferation [205]. Although, the rate of glycolysis varies on endothelial subtypes. Microvascular endothelial cells are known to be more glycolytic and proliferative [205, 211]. Despite generating very little ATP, endothelial cells are relied on less efficient glycolysis due to higher endothelial cell glucose uptake capability and faster ATP production [212, 213]. Endothelial cells use stored glycogen for energy production in vascular tissues [214, 215].

Although endothelial cells are not dependent on mitochondria for energy production, mitochondria act as biosynthetic hub endothelial growth and proliferation. Fatty acid oxidation sustains vessel growth. Stalk cells rely on fatty acid oxidation for their normal functions. Beta oxidation of fatty acids supports the DNA replication process. Shuttled long-chain fatty acids into mitochondria are metabolized to acetyl-CoA, used for the citric acid cycle. The use of fatty acid-derived carbon for nucleotide biosynthesis differentiates endothelial cells from glucose and glutamine-dependent proliferating cells [28, 216]. Maybe during a nutrition-deprived state, endothelial cells catabolize fatty acids from stored lipids. Growth and division, the development of new membrane, the generation of signaling molecules, and modulating cellular signaling, endothelial cells are solely dependent on fatty acids and other lipids [217]. To improve the intracellular lipid storage, oxaloacetate and acetyl-CoA genesis occur into the cytosol from the citric acid cycle derived citrate. Fatty acid synthase enzyme converts acetyl-CoA to fatty acids, which is crucial for angiogenesis and vascular homeostasis [26, 218].

Though, mitochondrial fatty acid biosynthesis diminishes mitochondrial integrity due to exhaust the citric acid cycle intermediates. Anaplerosis (the replenishing process of the citric acid cycle intermediates) can only prevent mitochondrial disintegration and cell death [212]. As an essential nitrogen source for nucleotide biosynthesis, endocytosed non-essential amino acid glutamine converts to glutamate and α-ketoglutarate to contribute to anaplerosis [219]. Glutaminase is the rate-limiting enzyme for the conversion of glutamine. Upregulation of glutaminase activity is apparent during angiogenesis [220]. Besides anaplerosis, glutamine can be used for energy production, biosynthesis of glutathione, and reductive carboxylation for lipid production [219, 221]. Prostanoids mediate angiogenesis through multiple mechanisms, including the induction of VEGF production [222], the stimulation of endothelial cell sprouting, migration, and tube formation [223–225].

Although extracellular-regulated kinase phosphorylated, MYC (V-Myc avian myelocytomatosis viral oncogene homolog) regulates endothelial metabolism and growth factor signaling for cell growth, proliferation, and anabolic metabolism [226, 227]. In addition, accumulated MYC increases glycolysis, mitochondrial function, and cell cycle progression [228]. Many studies focused on angiogenic endothelial cells; however, the metabolic role of quiescent endothelial cells is critical for normal vascular function. Transcription factor FOXO1 drives endothelial quiescence by reducing overall metabolic activity. In addition, FOXO1 suppresses MYC activity [226].

Interestingly, quiescent endothelial cells consistently reduce glucose uptake or glycolysis and increase fatty acid uptake [29]. The change in nutrient utilization or slower metabolic rate in quiescent endothelial cells needs to elucidate. Reduced metabolic activity helps stabilize vessels and ensures efficient nutrient and O2 delivery to perivascular tissues. Also, endothelial cells protect themselves from mitochondrial-derived ROS-mediated damage due to high oxygen levels in the bloodstream. So, it is convincible that the reduced metabolic rate of quiescent endothelial cells is a primary adaptive response.

Endothelial cells respond to changes in environmental conditions, e.g., hypoxia, glucose deprivation, and blood flow-mediated shear stress activates AMPK to promote catabolic pathways in endothelial cells by fatty acid oxidation to produce energy [169, 229, 230]. Besides, AMPK inhibits metabolic sensor mammalian targets of rapamycin complex 1 to promote endothelial cell migration and angiogenesis [231, 232]. In addition, another angiogenic regulator, sirtuin 1, also senses endothelial metabolic state [233].

## Metaflammation and ectopic fat deposition: effects on cardiovascular disease

Metabolic stress-induced chronic low-grade sterile inflammation due to innate immune response has been termed “metaflammation” [234]. Metaflammation is the critical driver of various chronic diseases, e.g., CVDs, diabetes, nonalcoholic steatohepatitis. In the body, inflammation is tightly controlled by distinct signaling cascades [235]. Pro-inflammatory mediators activate the innate immune system via various pattern recognition receptors [236, 237]. Most studies have focused on damage-associated molecular patterns (DAMPs) or pathogen-associated molecular patterns to distort the immune system. DAMPs have been found to link with the pathogenesis of chronic metabolic diseases or disorders [238]. But DAMPs are quite varied in structure and origin, and the list of DAMPs is enriching rapidly. Pattern recognition receptors sense free fatty acids, oxidized LDL, cholesterol crystals, glucose, advanced glycation end products. To delineate the inflammatory stimuli due to metabolic stress from other damage-associated immune responses, Wang et al. have used the fresh term “metabolic-associated molecular patterns” [239]. Molecules derived due to persistent excessive FFAs and glucose in plasma are considered to become metabolic-associated molecular patterns or so-called “DAMPs” for innate immune response mediated metaflammation [240, 241].

Pancreatic beta-cell dysfunction, metabolic syndrome, and insulin resistance are correlated with elevated plasma-FFAs [242–244]. Toll-like receptor (TLR)-2 or TLR-4 mediated production of interleukin-1β, tumor necrosis factor-α, macrophage inflammatory protein-1α, chemokine (C–C motif) ligand 2, and chemokine (C–C motif) ligand 4 are triggered by upregulated plasma saturated fatty acids [245–250].

**Fig. 4:**
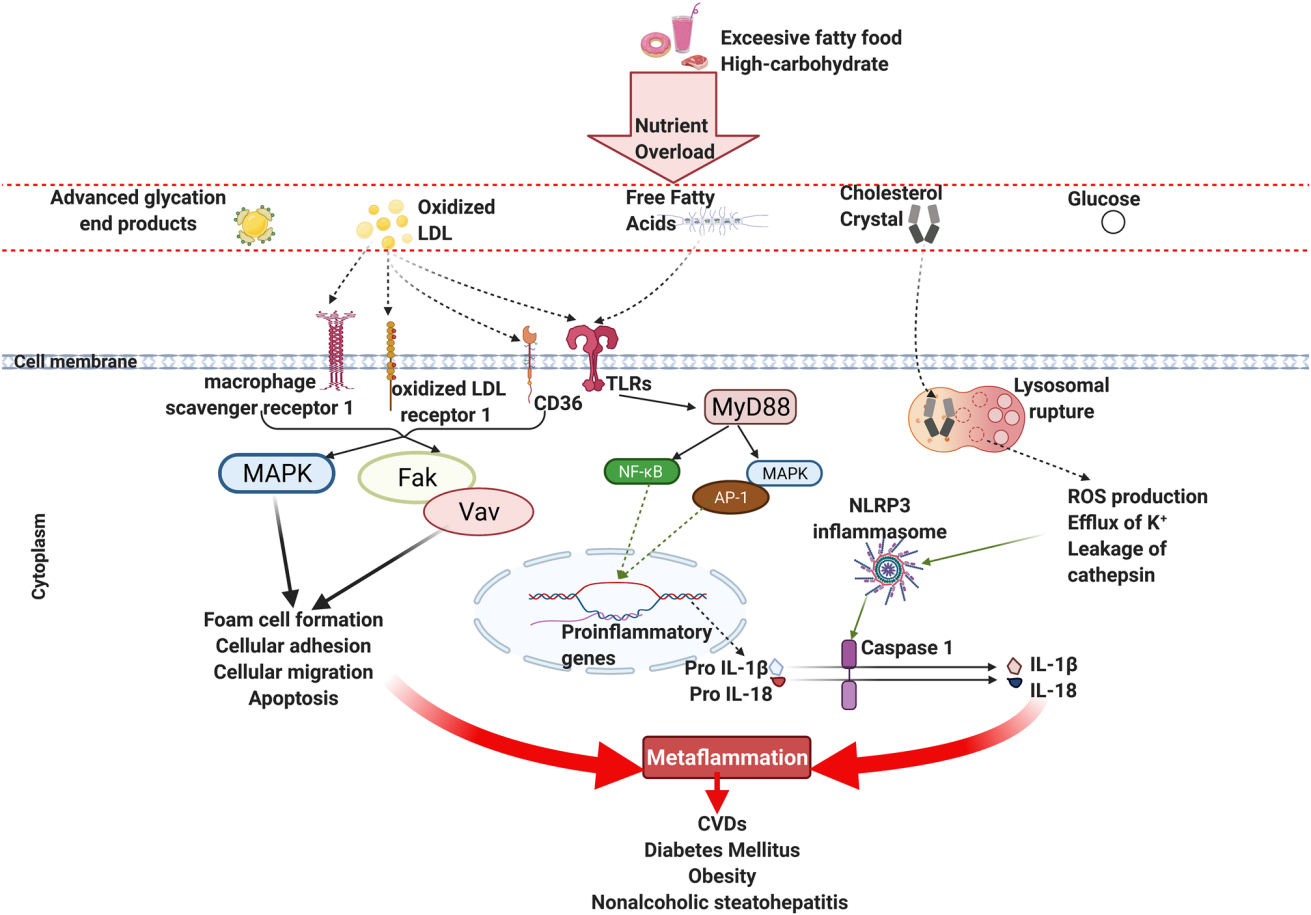
Metabolic-associated molecular patterns induce metaflammation. High glucose/fat causes an overload of nutrient metabolites, e.g., glucose, advanced glycation end products, free fatty acids, oxidized LDL, and cholesterol crystal. MAPK and canonical NF-κB pathway-mediated transcription of proinflammatory genes are driven by free fatty acids and/or oxidized LDL-induced TLRs. Transcription factors AP-1 and NF-κB are responsible for inciting the proinflammatory genes. Besides, scavenger receptors take oxidized LDL to form foam cells. Cholesterol crystals can damage the lysosome, which leads to ROS production, K^+^ efflux, and cathepsin leakage. These stimulatory factors activate the NLRP3 inflammasome and result in maturation and secretion of IL-1β and IL-18, thereby instigating metaflammation

Also, oxLDL induces pattern recognition receptors mediated chronic inflammation, which leads to various metabolic diseases or disorders, e.g., atherosclerosis, heart disease, hypertension, obesity [251–254]. 80% oxLDL is taken up by transmembrane scavenger receptors (macrophage scavenger receptor 1, CD36, and lectin-type ox LDL receptor 1) to activate different intracellular signaling pathways, e.g., NF-κB and MAPK [255–257]. Figure 4 shows the metabolic-associated molecular patterns that induce metaflammation. Even oxLDL can form immune complexes with antibodies to activate NLPR3 inflammasome, extracellular signal-regulated kinase 1/2, phospholipase C gamma, c-Jun N-terminal kinase, paxillin, spleen tyrosine kinase, and cell division control protein 42 homolog signaling by Fc gamma receptor, CD36, and/or TLR-4 [258, 259]. Like oxLDL, oxidized phospholipids in platelet induce CD36/TLR- 2/TLR-6 complex-mediated MyD88 signaling in the progression of atherothrombosis [260].

**Fig. 5:**
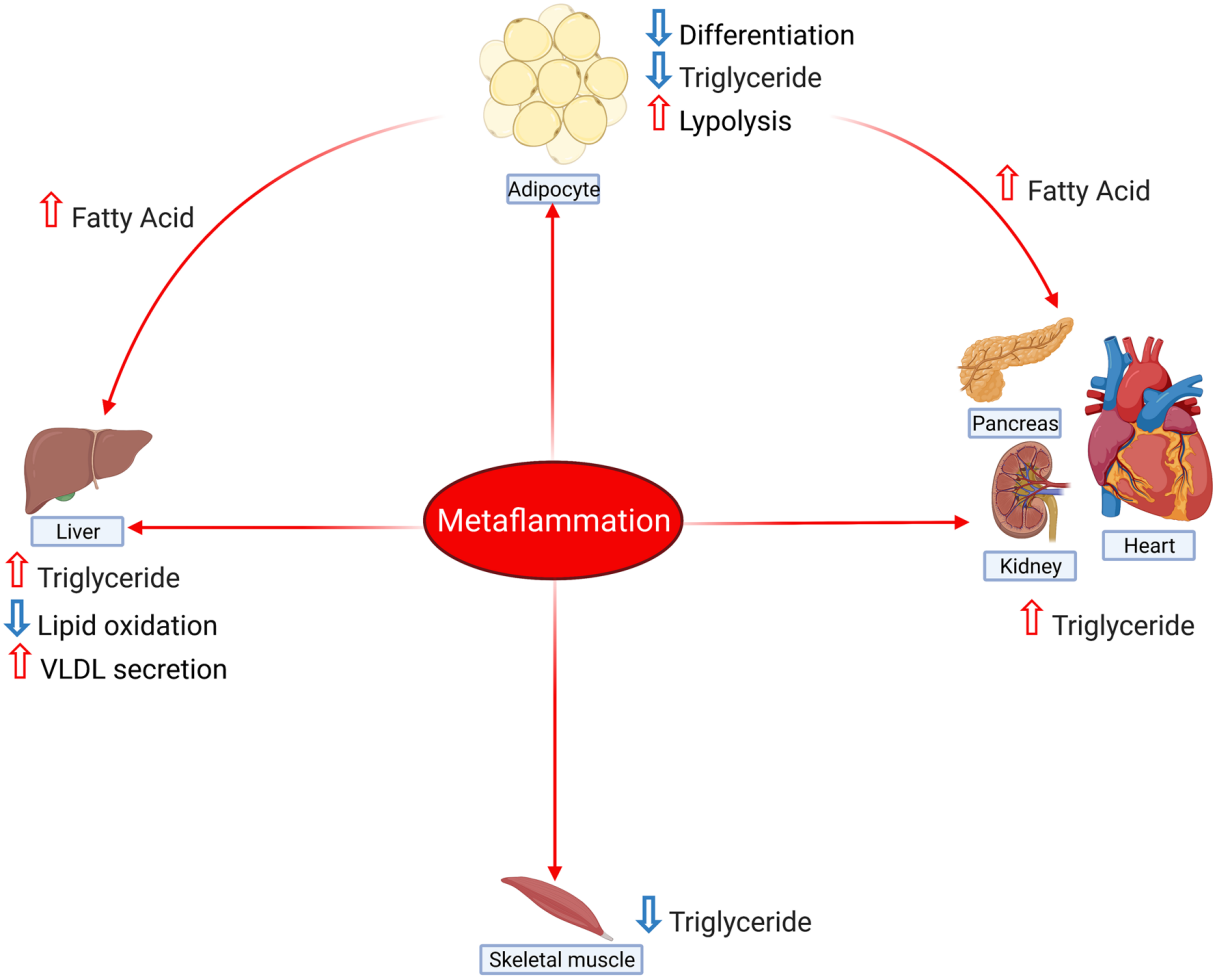
Effect of metaflammation on different organs. Metaflammation-induced cytokines to increase lipid deposition in the liver, heart, kidney, and pancreas, while lipolysis and fatty acid production are evident in adipose tissue. Similarly, triglyceride content reduces in skeletal muscle following chronic low-grade inflammation

Inflammation provokes lipolysis in adipocytes and increases serum FFAs [261, 262]. Similar to adipose tissue, inflammation negatively regulates lipid deposition in skeletal muscle [263]. However, TNFα doesn’t affect fatty acid oxidation but upregulates fatty acid incorporation with diacylglycerol [264–266]. Figure 5 summarises the effects of metaflammation in different organs.

As the prime metabolic organ, de novo* lipogenesis* (DNL), an influx of FFAs from adipose tissue due to lipolysis, fatty acid oxidation, and VLDL secretion maintain hepatic lipid homeostasis [267]. In addition, inflammation is associated with overexpression of FAT/CD36 and FABPs in the liver [268–270]. Systemic inflammation also increases hepatic de novo lipid synthesis by upregulation of several genes such as sterol regulatory element-binding protein 1c (*SREBP-1c), FAS, ACC*, and *SCD-1,* which can be ameliorated by sirtuin1 [271, 272]. More research is required to understand the mechanism of inflammation and its effects on non-adipose tissues.

## Conclusions

The endothelium transcends key regulators to the function of every organ system. Endothelium dysfunction can lead to many diseases. Elevated levels of FFAs due to the result of metabolic defects contribute to endothelium dysfunction and subsequently lead to many diseases such as CVD. Dysfunction endothelium develops decreased NO production, increased cytokine production, impaired vasodilation, hyperactivity of platelets, and increased oxidative stress and inflammation. Consuming foods rich in n-3 fatty acids can lower plasma FFAs and other lipids with associated inflammatory cytokines, oxidative stress and may protect the endothelium. However, it would require a better understanding of FFAs and their transport and actions in endothelium and identify some better possible targets that could be used to develop better therapeutic approaches to intervene in the early events endothelium dysfunction.

## Abbreviations

ACE: Angiotensin-converting enzyme
ANGPT2: Angiopoietin 2
ANGPTL4: Angiopoietin-like 4
COX: Cyclooxygenase
CVD: Cardiovascular disease
DAMP: Damage-associated molecular pattern
DHA: Docosahexaenoic acid
EPA: Eicosapentaenoic acid
ET-1: Endothelin-1
EPC: Endothelial progenitor cell
FFA: Free fatty acid
FABP: Fatty acid-binding protein
CD36: Cluster of differentiation 36
FATP: Fatty acid transport proteins
LCFA: Long-chain saturated fatty acid
LCPUFA: Long-chain polyunsaturated fatty acid
LDL: Low-density lipoprotein
MYC: V-Myc avian myelocytomatosis viral oncogene homolog
NO: Nitric oxide
PPARα: Peroxisome proliferator-activated receptor alpha
PPARγ: Peroxisome proliferator-activated receptor-gamma
PG: Prostaglandin
PUFA: Polyunsaturated fatty acid
RAS: Renin-angiotensin system
ROS: Reactive oxygen species
SREBP-1c: Sterol regulatory element-binding protein 1c
SERCA: Sarco/endoplasmic reticulum calcium ATPase
Tx: Thromboxane
TNFα: Tumor necrotic factor α
TLR: Toll-like receptor
